# Particulate-phase mercury emissions from biomass burning and impact on resulting deposition: a modelling assessment

**DOI:** 10.5194/acp-17-1881-2017

**Published:** 2017

**Authors:** Francesco De Simone, Paulo Artaxo, Mariantonia Bencardino, Sergio Cinnirella, Francesco Carbone, Francesco D’Amore, Aurélien Dommergue, Xin Bin Feng, Christian N. Gencarelli, Ian M. Hedgecock, Matthew S. Landis, Francesca Sprovieri, Noriuki Suzuki, Ingvar Wängberg, Nicola Pirrone

**Affiliations:** 1 CNR-Institute of Atmospheric Pollution Research, Division of Rende, UNICAL-Polifunzionale, 87036 Rende, Italy; 2 University of Sao Paulo, Sao Paulo, Brazil; 3 Univ. Grenoble Alpes, CNRS, IRD, IGE, Grenoble, France; 4 Institute of Geochemistry, State Key Laboratory of Environmental Geochemistry, Chinese Academy of Sciences, Guiyang, China; 5 Office of Research and Development, US Environmental Protection Agency, Research Triangle Park, NC, USA; 6 National Institute for Environmental Studies (NIES), Ministry of Environment, Okinawa, Japan; 7 IVL, Swedish Environmental Research Inst. Ltd., Göteborg, Sweden; 8 CNR-Institute of Atmospheric Pollution Research, Area della Ricerca di Roma 1, Via Salaria km 29 300, Monterotondo, 00015 Rome, Italy

## Abstract

Mercury (Hg) emissions from biomass burning (BB) are an important source of atmospheric Hg and a major factor driving the interannual variation of Hg concentrations in the troposphere. The greatest fraction of Hg from BB is released in the form of elemental $\text{Hg}\;({\text{Hg}}_{\left(g\right)}^{0})$. However, little is known about the fraction of Hg bound to particulate matter (Hg^P^) released from BB, and the factors controlling this fraction are also uncertain. In light of the aims of the Minamata Convention to reduce intentional Hg use and emissions from anthropogenic activities, the relative importance of Hg emissions from BB will have an increasing impact on Hg deposition fluxes. Hg speciation is one of the most important factors determining the redistribution of Hg in the atmosphere and the geographical distribution of Hg deposition. Using the latest version of the Global Fire Emissions Database (GFEDv4.1s) and the global Hg chemistry transport model, ECHMERIT, the impact of Hg speciation in BB emissions, and the factors which influence speciation, on Hg deposition have been investigated for the year 2013. The role of other uncertainties related to physical and chemical atmospheric processes involving Hg and the influence of model parametrisations were also investigated, since their interactions with Hg speciation are complex. The comparison with atmospheric Hg^P^ concentrations observed at two remote sites, Amsterdam Island (AMD) and Manaus (MAN), in the Amazon showed a significant improvement when considering a fraction of Hg^P^ from BB. The set of sensitivity runs also showed how the quantity and geographical distribution of Hg^P^ emitted from BB has a limited impact on a global scale, although the inclusion of increasing fractions Hg^P^ does limit ${\text{Hg}}_{\left(g\right)}^{0}$ availability to the global atmospheric pool. This reduces the fraction of Hg from BB which deposits to the world’s oceans from 71 to 62 %. The impact locally is, however, significant on northern boreal and tropical forests, where fires are frequent, uncontrolled and lead to notable Hg inputs to local ecosystems. In the light of ongoing climatic changes this effect could be potentially be exacerbated in the future.

## 1 Introduction

Emissions from biomass burning (BB) are an important source of mercury (Hg) to the atmosphere (De Simone et al., 2015; Friedli et al., 2009) and a major factor in determining the interannual variations of its tropospheric concentration (Slemr et al., 2016). Although the Hg released by BB varies from year to year, it can amount to up to roughly one third of the anthropogenic emission estimates (AMAP/UNEP, 2013; Friedli et al., 2009; De Simone et al., 2015). With the eventual implementation of the Minamata Convention (http://www.mercuryconvention.org/) and future curbs on industrial emission, as a by-product of industrial emission abatement measures, its relative importance will increase in the coming years. A previous modelling study (De Simone et al., 2015) used the global Hg chemistry model, ECHMERIT, and three BB inventories to assess the distribution of Hg deposition resulting from BB. A large part of the Hg released from BB deposits over oceans, where its re-emission is driven by sea surface temperature, among other factors (Carbone et al., 2016; Andersson et al., 2011), or where it can be converted to toxic methyl mercury (MeHg) compounds, has important implications for the food web and, through fish consumption, also for human health (see Chen et al., 2016, and references therein). The deposition flux of Hg from BB has been shown to be more sensitive to certain factors, in particular the chemical mechanism employed in the model and the choice of emission inventory, than to others such as the vertical profiles of emissions (De Simone et al., 2015). In this previous study all Hg emitted from BB was considered to be ${\text{Hg}}_{\left(g\right)}^{0}$. There is, however, evidence that the fraction of Hg emitted bound to particulates (Hg^P^) may be sizeable, up to 30 %, especially when the fuel moisture content (FMC) is high (Obrist et al., 2007; Finley et al., 2009; Friedli et al., 2009; Wang et al., 2010). These levels, however, remain uncertain since different methodologies have led to different conclusions (Zhang et al., 2013; Obrist et al., 2007). Little is known about the mechanisms that control the speciation of Hg in BB emissions, which leads to uncertainties in the Hg deposition patterns, since the atmospheric lifetime of Hg^P^ is significantly shorter than ${\text{Hg}}_{\left(g\right)}^{0}$, leading to greater local deposition. Local Hg deposition due to BB could have important repercussions in regions such as the South-East Asia, where there is intensive rice cultivation, which is subject to major BB events, especially during El Niño periods. Hg deposited to rice paddies can be readily converted to toxic MeHg that can accumulate in the grains (Wang et al., 2015; Feng et al., 2008; Meng et al., 2014; Zhang et al., 2010). Moreover, it has been reported that Hg^P^ from BB deposited to foliage has the ability to enhance MeHg formation (Witt et al., 2009). The aim of this study is to investigate the effects on simulated deposition fluxes of Hg resulting from BB when variations in Hg^P^ fraction and production processes are considered. The most recent version of the GFED BB emission inventory (van der Werf et al., 2010; Randerson et al., 2012; Mu et al., 2011), has been included in the global online Hg chemical transport model ECHMERIT to simulate Hg deposition from BB for the year 2013 and to quantify the influence of variations in model inputs, assumptions and parametrisations.

## 2 Methods

### 2.1 The biomass burning inventory

The reference BB inventory in this study, Global Fire Emissions Database version 4 (GFED4.1s), is based on an updated version of the inventory of van der Werf et al. (2010) with burned area from Giglio et al. (2013), and with the addition of small fire-burned area (Randerson et al., 2012). The standard temporal resolution of the emissions files is monthly, but data are provided to distribute these daily, and a diurnal cycle based on Mu et al. (2011) is also available. Daily BB emissions from two other global inventories, GFASv1.2 (Kaiser et al., 2012, 2015) and FINNv1.5 (Wiedinmyer et al., 2011), were also included in the model for sensitivity runs. These three inventories are all compiled using the imagery obtained from the MODIS instruments. However, the way in which the data are filtered or processed yields substantial differences between the final products; see Andela et al. (2013) and references therein for a detailed description of the differences among the inventories.

### 2.2 Experimental set-up

The global Hg chemical transport model ECHMERIT (Jung et al., 2009; De Simone et al., 2014) uses T42 horizontal resolution (roughly 2.8° by 2.8° at the Equator) and 19 vertical levels up to 10 hPa. Hg emissions from BB were included in the model by mapping them to CO emissions using the global averaged enhancement ratio (ER) of 1.96 × 10^−7^, as obtained by Friedli et al. (2009), averaging field measurements from different biomes in various regions around the globe, including in plume measurements from the CARIBIC project (Ebinghaus et al., 2007). Previous modelling studies have used different ERs (De Simone et al., 2015; Holmes et al., 2010), but all these values were well within the range of uncertainty (0.3–6.0× 10^−7^; see Wang et al., 2015). ECHMERIT, in the base configuration, includes the oxidation of ${\text{Hg}}_{\left(g\right)}^{0}$ to in ${\text{Hg}}_{\left(g/\text{aq}\right)}^{\text{II}}$ oxidation by O3 / OH in the gas and aqueous phases. OH and O_3_ concentration fields were imported from MOZART (Model for Ozone and Related chemical Tracers) (Emmons et al., 2010). Hg^P^ is assumed to be inert, whether it is emitted from anthropogenic activities or BB, and it is subject to transport and deposition processes but is not involved in any chemical reactions. Mechanisms and parametrisations used for calculating the dry and the wet deposition of the different Hg species are the same as described in Jung et al. (2009). Beyond this standard configuration a number of alternative processes and chemical mechanisms have been considered for this study, as explained in Sect. 2.3. Atmospheric reduction of ${\text{Hg}}_{\left(g/\text{aq}\right)}^{\text{II}}$ to ${\text{Hg}}_{\left(g\right)}^{0}$ has been included in many models to regulate the residence time of ${\text{Hg}}_{\left(g\right)}^{0}$ in the atmosphere. However, a number of the proposed mechanisms are unlikely to occur under most atmospheric conditions or are based on empirical rates to better match the observations (see Kwon and Selin (2016) for a recent review). Due to this uncertainty, reduction was not included in this study. No further Hg^P^ particulate matter (PM) dimension distributions other than the standard log-normal particle size distribution, as described in detail in (Jung et al., 2009), were considered in this study due to large uncertainties regarding the dynamic size range of PM emitted during BB (see Janhäll et al. (2010) and references therein). GFED4.1s provides monthly burned area, fire carbon (C) and dry matter (DM) emissions (http://www.falw.vu/~gwerf/GFED/GFED4/). A script is provided to derive gaseous and PM emissions from DM fields making use of biome-based emission factors based on Akagi et al. (2011) and van der Werf et al. (2010). The resulting emission fields were then interpolated on to the ECHMERIT T42 grid using the mass conserving remapping function included in the Climate Data Operators (https://code.zmaw.de/projects/cdo).

### 2.3 Simulations and their scope

The BASE simulation used as the reference case in this study includes daily BB emissions from GFEDv4.1s, in which a global uniform fraction of Hg^P^, equal to 15 % of the total Hg emission is assumed. This value is within the range of observations (Obrist et al., 2007; Finley et al., 2009). However, since there are uncertainties in the proportion of Hg^P^ emitted from BB (Zhang et al., 2013), further simulations were carried out with varying fractions of Hg^P^ (0, 4 and 30 %). Simulations were also conducted mapping the 15 % of the total Hg emitted as Hg^P^ to the geographical distribution of different proxy chemical species (see Sect. 2.4). The shorter lifetime of Hg^P^ with respect to ${\text{Hg}}_{\left(g\right)}^{0}$ potentially means that the vertical profile of the emissions could have an impact on the distribution of Hg deposition, as is the case for other speciated Hg emission sources (De Simone et al., 2016). Therefore two vertical profile parametrisations, as well as different emission injection time resolutions, were also included in the study. The principal vertical profile used (PBL-Profile) maps the Hg emissions uniformly within the planetary boundary layer (PBL), whereas in the second the vertical profile of the standard version of the ECHAM-HAM model was used (HAM-Profile) (Zhang et al., 2012). The HAM-Profile is equal to PBL-Profile when the PBL height is greater than 4000 m; otherwise 75 % of the emissions are placed within the PBL and the remainder in the two layers above the PBL (17 and 8 %). This threshold value is arbitrary, but it is the standard configuration of ECHAM6-HAM2 (Zhang et al., 2012; Veira et al., 2015). Biomass burning emissions from GFASv1.2 (Kaiser et al., 2012, 2015) and FINNv1.5 (Wiedinmyer et al., 2011) were also used in the study to assess uncertainty related to the satellite imagery processing and inventory compilation. Simulations using GFASv1.2 were excluded from suqsequent analyses since the low Hg emissions could be due to a technical problem arising from GRIB encoding (see GFAS, 2015). These simulations primarily employ a $O_{3}/\text{OH}\;{\text{Hg}}_{\left(g\right)}^{0}$ oxidation mechanism. However, since the precise atmospheric Hg oxidation mechanism remains unclear (Hynes et al., 2009; Subir et al., 2011, 2012; Gustin and Jaffe, 2010; Gustin et al., 2015; Ariya et al., 2015), a number of runs were performed using a Br-based oxidation mechanism. Some studies (Steffen et al., 2014; Amos et al., 2012) suggest that the partitioning of reactive Hg species the between gas and particulate phases might be driven by air temperature and on the surface are of the aerosol present in the atmosphere. Therefore, two other simulations were conducted including the temperature-dependent gas-particle partitioning described in Amos et al. (2012), one assuming BB Hg emissions to be only ${\text{Hg}}_{\left(g\right)}^{0}$ and another assuming a 15 % of BB Hg emissions to be Hg^P^. To estimate the ratio of Hg deposition from BB compared to anthropogenic sources, six further simulations were conducted including only anthropogenic emissions using the EDGAR (Muntean et al., 2014), AMAP2010 (AMAP/UNEP, 2013) and STREETS (Corbitt et al., 2011) inventories, employing the O_3_ / OH and Br oxidation mechanisms. This study covers a single year, 2013, chosen due to the availability of measurements from GMOS network (Sprovieri et al., 2016a, b; D’Amore et al., 2015). All simulations were performed for a full year, without the rapid re-emission mechanism (Selin et al., 2008), and were continued without further emissions for another 12 months to allow most of the 2013 Hg emissions to be deposited. Finally, a selection of simulations were re-run including Hg emissions from all sources, BB, anthropogenic emissions from AMAP2010 (AMAP/UNEP, 2013), dynamic ocean emissions, terrestrial emissions and re-emissions as described in De Simone et al. (2014), to evaluate model performance against measurements and to evaluate the assumptions made in this study. A summary of the simulations performed can be found in Table 1.

### 2.4 BB emission speciation

**Figure 1:**
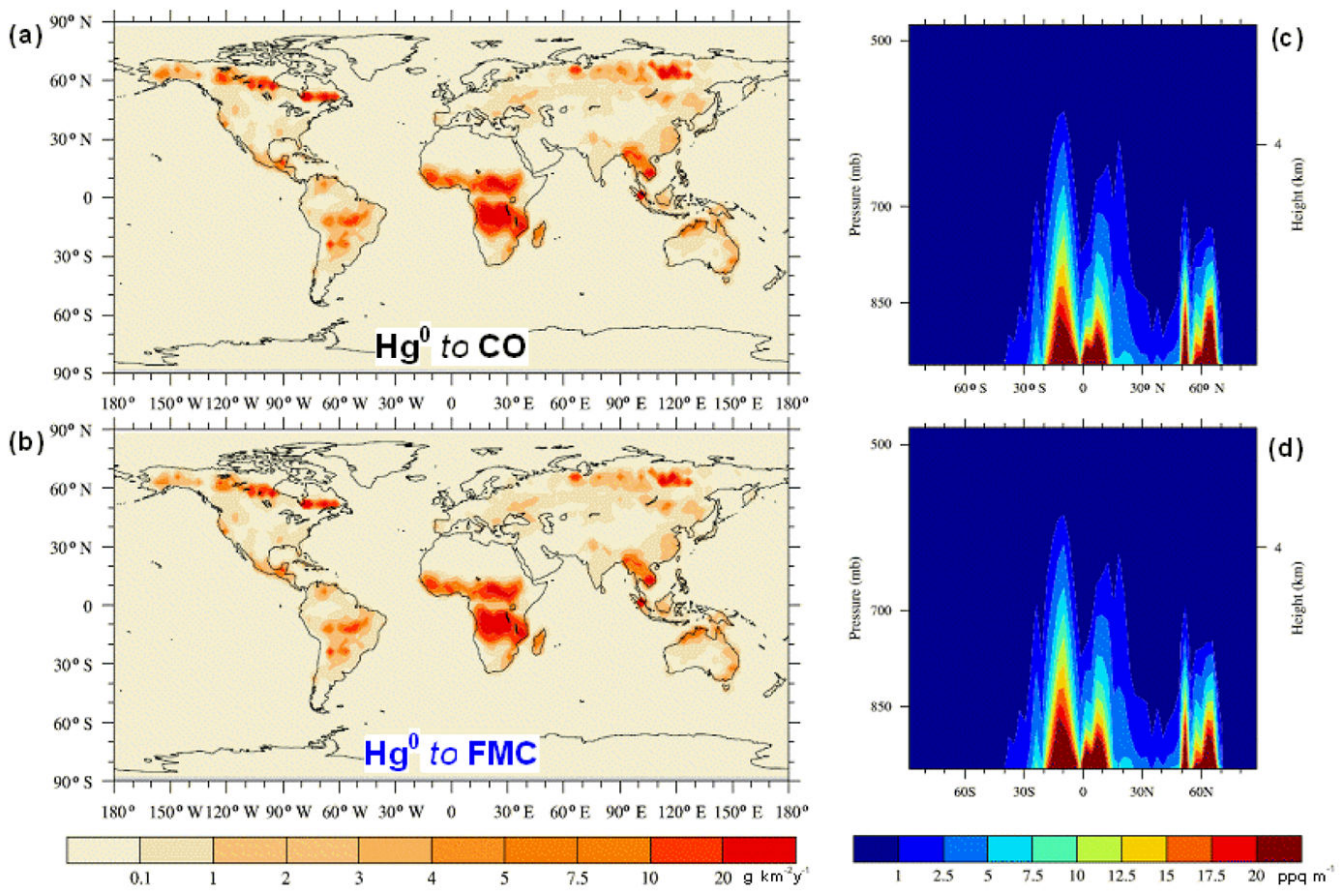
Geographical distribution **(a–b)** and PBL-type vertical profiles **(c–d)** of the ${\text{Hg}}_{\left(g\right)}^{0}$ emissions, when mapped to CO **(a, c)** and when speciation is determined by FMC **(b, d)**. For the emissions mapped to CO, only the speciation ( $15:85\;{\text{Hg}}^{P}:{\text{Hg}}_{\left(g\right)}^{0}$) is shown for clarity.

**Figure 2:**
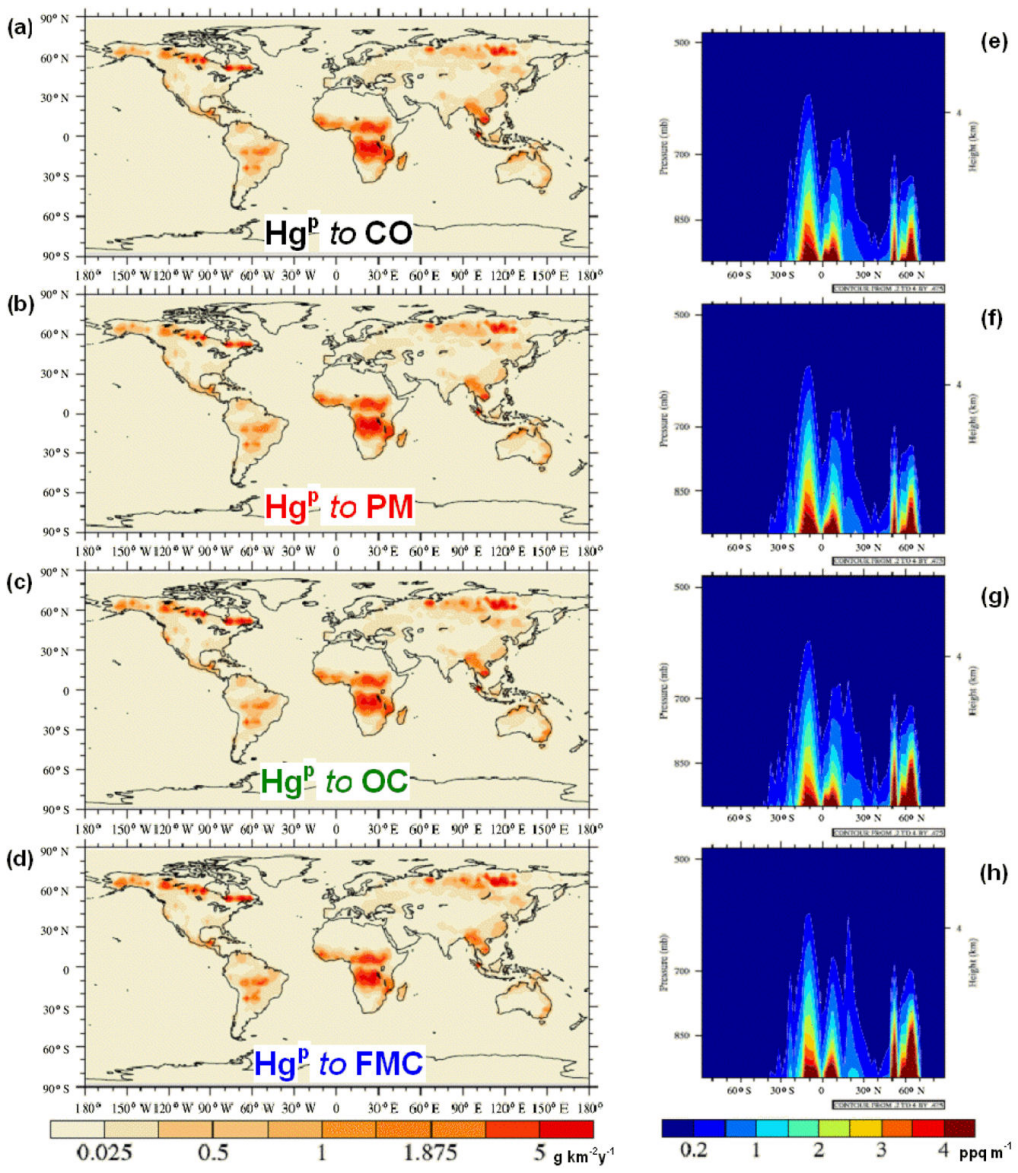
Geographical distribution **(a–d)** and PBL-type vertical profiles **(e–h)** of the Hg^P^ emissions as injected in the model, when mapped to CO **(a, e)**, PM **(b, f)** and OC **(c, g)** and when speciation is determined by FMC **(d, h)**. For the emissions mapped to CO, only the speciation ( $15:85\;{\text{Hg}}^{P}:{\text{Hg}}_{\left(g\right)}^{0}$) is shown for clarity.

The release of Hg from BB occurs prevalently as ${\text{Hg}}_{\left(g\right)}^{0}$. However, as mentioned previously, a measurable fraction may be emitted as Hg^P^ (Obrist et al., 2007; Friedli et al., 2009; Finley et al., 2009; Wang et al., 2010). No significant amounts of gaseous oxidised $\text{Hg}\;({\text{Hg}}_{\left(g\right)}^{\text{II}})$ have so far been detected in BB emissions (Obrist et al., 2007, and references therein). The speciation of Hg emissions is of great importance, since it largely determines the atmospheric lifetime and hence the distance emitted Hg is transported in the atmosphere before deposition, as seen for other speciated Hg sources (Bieser et al., 2014). The fraction of Hg^P^ released by BB determined in field and laboratory studies ranges from fractions of a few percent to over 30 % (Obrist et al., 2007). The factors determining speciation and whether Hg^P^ is directly emitted or if it is the product of the oxidation of ${\text{Hg}}_{\left(g\right)}^{0}$ within the plume (Obrist et al., 2007; Webster et al., 2016) are not known. However, foliage, moisture content, fuel type, plant species and combustion proprieties certainly play a role. Hg^P^ emissions were found to be well correlated with particulate matter (PM) and organic carbon (OC) emissions (Obrist et al., 2007). Obrist et al. (2007) found that ${\text{Hg}}_{\left(g\right)}^{0}$ is the dominant species in dry fuel combustion, whereas the fraction of Hg^P^ becomes appreciable when FMC reaches roughly 30 %, above which Hg^P^ release appears to increase linearly with FMC. In the inventory used for the BASE case both ${\text{Hg}}_{\left(g\right)}^{0}$ and Hg^P^ follow the spatial distribution of CO emissions from BB, and 15 % of the emitted Hg is considered to be Hg^P^ (see Figs. 1a and 2a). Hg emission fields were also compiled in which the Hg^P^ fraction of the total Hg emitted was mapped to OC and PM emissions (see Fig. 2b and c). A further emission field was compiled in which the ratio of ${\text{Hg}}_{\left(g\right)}^{0}$ to Hg^P^ is determined by the FMC (Figs. 1b and 2d). A relationship was found to exist between Hg^P^ emissions and the fire burn duration and severity as well as combustion conditions (Obrist et al., 2007; Webster et al., 2016). In particular high Hg^P^ fractions were observed during smouldering phases, whereas very low or undetectable Hg^P^ levels were found during flaming combustion. These potential parametrisations were not investigated here due to the difficulty in finding a suitable proxy data set. Appendix A contains a more detailed description of the methods used to calculate the different Hg BB emission fields.

## 3 Results

### 3.1 Emissions

**Figure 3:**
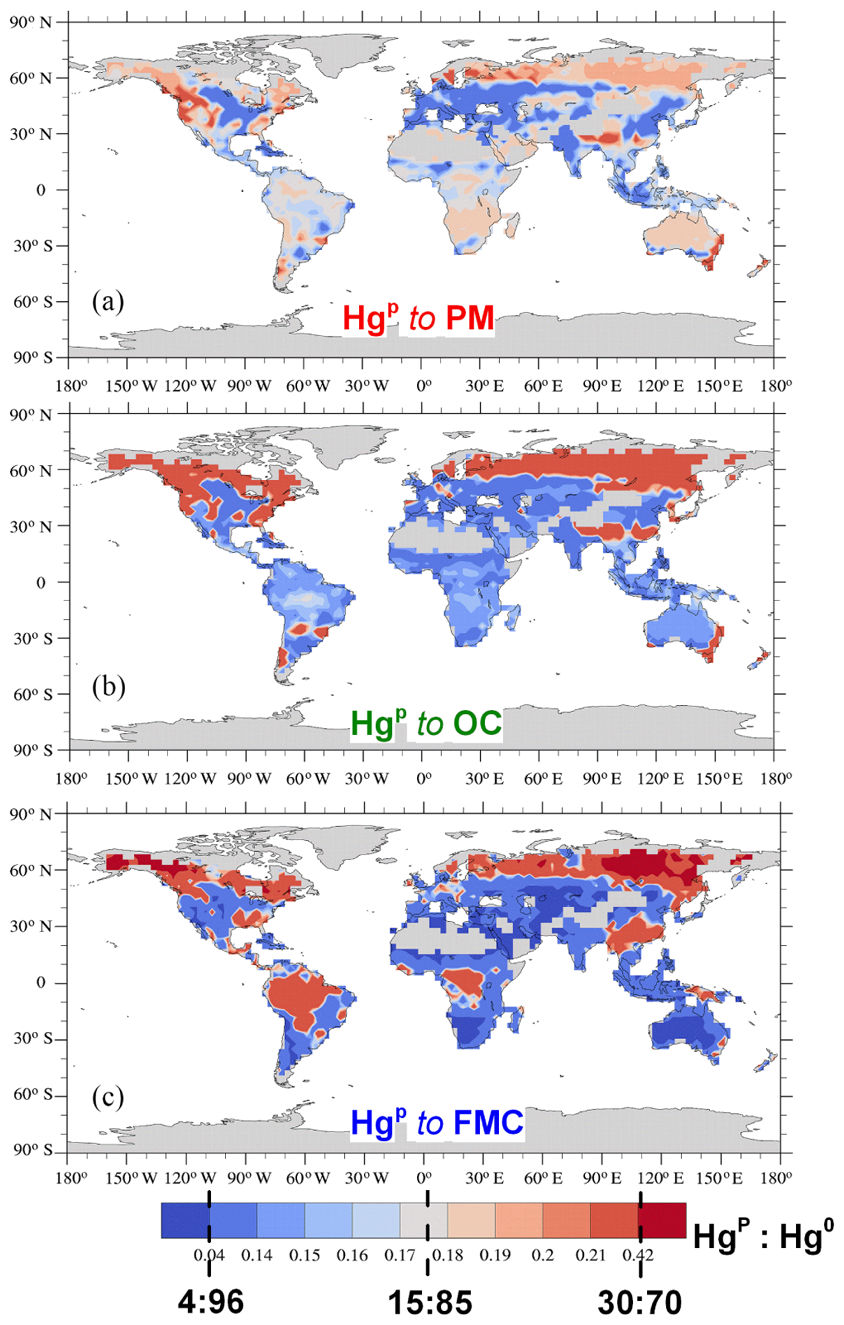
Geographical distribution of the ${\text{Hg}}^{P}:{\text{Hg}}_{\left(g\right)}^{0}$ emissions ratio, when mapped to PM **(a)** and OC **(b)** and when speciation is determined by FMC **(c)**. In the colour bar the levels corresponding to the constant speciations (4 : 96, 15 : 85 and $30:70\;{\text{Hg}}^{P}:{\text{Hg}}_{\left(g\right)}^{0}$) used for the emissions mapped to CO are indicated.

The total Hg emitted in 2013 based on the GFED inventory is roughly 400 Mg, which is at the lower end of the initial estimates (675 ± 240 Mg) (Friedli et al., 2009) but is reasonable considering the natural variation of BB activity and the diminishing trend of the CO emission estimates in the latest inventory revisions (up to 50 % for some years) (van der Werf et al., 2010). Considering 15 % of the emissions to be Hg^P^, in the BASE run this corresponds to approximately $340\;\text{Mg}\;{\text{Hg}}_{\left(g\right)}^{0}$ and 60 Mg Hg^P^. Interestingly the emissions of Hg^P^ amount to 58 Mg when relating the Hg^P^ fraction to FMC. The exact amount of Hg emitted by BB in the different model runs is detailed in Table 1. The spatial distribution and the vertical profile of the emission injection height, considering the PBL-Profile for ${\text{Hg}}_{\left(g\right)}^{0}$ and Hg^P^ in the different cases considered are shown in Figs. 1 and 2. Both the geographical and vertical distributions of the emissions of the Hg species reveal notable differences depending on the methodology used, particularly for Hg^P^. Compared to the cases where Hg^P^ emissions are mapped to CO and PM (Fig. 2a–b and e–f), mapping Hg^P^ to OC and using the FMC to determine the speciation (Fig. 2c–d and g–h) result in enhanced Hg^P^ emissions, above 60° N and over some areas the Amazon, central Africa and East Asia as evident in Fig. 3. The timing and location of the enhanced Hg^P^ emission at northerly latitudes could be particularly relevant for Hg deposition to the Arctic. From Fig. 3 it is evident how the geographical distribution of the Hg^P^ to ${\text{Hg}}_{\left(g\right)}^{0}$ emission ratio differs with the assumptions considered. However, for OC and FMC there is general agreement on the areas where the Hg^P^ emissions are relatively higher, especially in the Northern Hemisphere and particularly for areas above 60° N. The agreement between OC and FMC is not surprising and is related to the combustion characteristics that enhance OC emissions, i.e. lower combustion temperatures and the dominance of the smouldering phase of combustion (Zhang et al., 2013), that are likely to occur where FMC is greatest.

### 3.2 Emission latitudinal profiles

**Figure 4:**
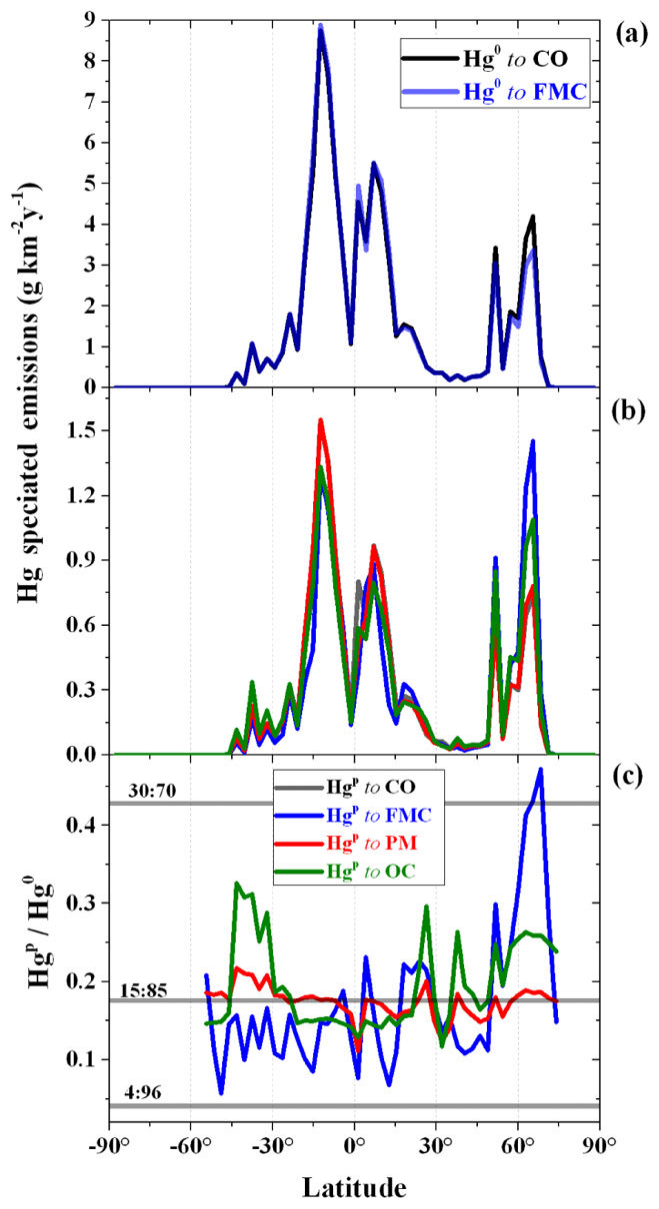
Latitudinal profiles of **(a)** ${\text{Hg}}_{\left(g\right)}^{0}$ emissions when mapped to CO and when speciation is determined by FMC; **(b)** Hg^P^ emissions when mapped to CO, PM and OC and when speciation is driven by FMC; and **(c)** the relevant ratio ${\text{Hg}}^{P}:{\text{Hg}}_{\left(g\right)}^{0}$. For both ${\text{Hg}}_{\left(g\right)}^{0}$ and Hg^P^ emissions mapped to CO, only the speciation ( $15:85\;{\text{Hg}}^{P}:{\text{Hg}}_{\left(g\right)}^{0}$) is reported for clarity, whereas in **(c)** all the speciations are reported.

**Figure 5:**
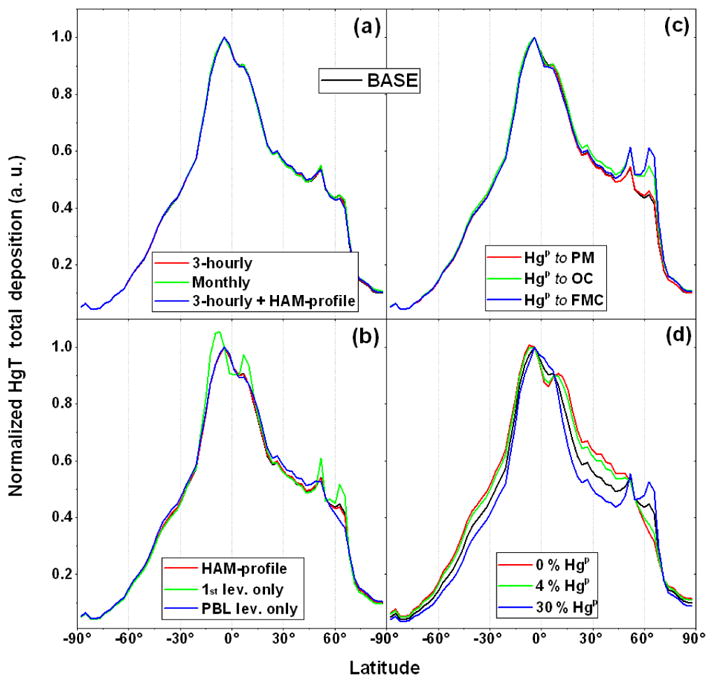
Latitudinal profiles of the normalised Hg total deposition from the model BASE run, compared with a selection of sensitivity runs, assuming **(a–b)** different emission time resolution and vertical profile, as well as a combination of both; **(c)** different Hg^P^ emission geographical distributions, as well as different ${\text{Hg}}_{\left(g\right)}^{0}:{\text{Hg}}^{P}$ ratios. The normalisation was done by maximum.

The latitudinal profiles of ${\text{Hg}}_{\left(g\right)}^{0}$ and Hg^P^ emissions, using the different approaches (Sect. 2.4), are shown in Fig. 4a and b. For those emissions mapped to CO, only the $15:85\;({\text{Hg}}^{P}:{\text{Hg}}_{\left(g\right)}^{0})$ speciation is reported for clarity. The differences in the latitudinal profiles of the ${\text{Hg}}_{\left(g\right)}^{0}$ emissions (Fig. 4a) are sizeable only for the peaks north of 45° N, where the FMC-based speciation has an ${\text{Hg}}_{\left(g\right)}^{0}$ fraction below 85 %. The latitudinal profiles of Hg^P^ emissions mapped to PM and CO look very similar over the entire domain (Fig. 4b), apart from a peak a few degrees north of the Equator. The Hg^P^ emissions mapped to OC and FMC differ from the PM and CO profiles but are similar to each other between roughly 30° S and 60° N. South of 30° S Hg^P^ emissions mapped to OC are higher, while peak Hg^P^ emissions derived from FMC at 65° N (1.5 g km^−2^ yr^−1^) are nearly 30 % greater than those derived from OC and roughly double those mapped to CO and PM. Moreover, in the FMC scenario the peak in Hg^P^ emissions at 65° N are greater than the peak seen at 15° S (1.5 vs. 1.4 g km^−2^ yr^−1^). As is particularly evident in Fig. 4c, the most notable differences among the different assumptions hypothesised are above 60° N, where both the OC and the FMC cases agree on the location of the greatest Hg^P^ emissions probably due to the linkage between OC emissions and combustion processes favoured by FMC (Zhang et al., 2013), and between 30 and 45° S, where only OC and PM are greater than BASE. A previous modelling study focusing on the fate of Hg from BB, where all emissions were considered as ${\text{Hg}}_{\left(g\right)}^{0}$, showed that the long atmospheric life of the elemental Hg smoothed the deposition latitudinal profiles compared to the emission profiles (De Simone et al., 2015). The four panels in Fig. 5 compare the normalised latitudinal deposition profiles obtained for the BASE simulation with those obtained from the alternative Hg^P^ emission scenarios by category. Figure 5a demonstrates the very limited impact of the time resolution used for BB emissions, most likely due to the coarse horizontal resolution of the model. The two vertical emission profiles (Fig. 5b) give deposition fields that are to all effects indistinguishable, even when considering varying temporal resolution of the BB emissions, whereas assuming all emissions to be in the first model level (with an average height of approximately 35 m) leads to enhanced deposition near emission peaks. In this instance, the maximum deposition coincides with peak emission, at approximately 15° S, whereas in all other cases maximum deposition is shifted towards the Equator.

The similarities in the latitudinal profiles of Hg^P^ emissions when mapped to CO and PM are reflected in their deposition profiles (Fig. 5c). The relatively greater deposition north of 60° N seen in Fig. 5c, obtained when Hg^P^ emissions are mapped to OC and when driven by FMC, reflects the peak in Hg^P^ emissions at this latitude. The greatest differences in the latitudinal deposition profiles, using the GFED inventory, are seen when varying the percentage of Hg^P^ in the emissions (Fig. 5d). Considering emissions to be solely ${\text{Hg}}_{\left(g\right)}^{0}$ yields a relatively smooth profile extending from pole to pole, increasing Hg^P^ causes enhanced deposition near BB hotspots. The emission peak at around 50° N remains relatively distinct also in the deposition for all the simulations (although it seen as a shoulder in the $100\;\%\;{\text{Hg}}_{\left(g\right)}^{0}$ profile). The peak north of 60° N is more dependent on emission speciation, supporting the previous finding that the location of Hg deposition depends on complex interactions between emission location and the time of year which influences both atmospheric transport patterns and oxidant concentration fields (De Simone et al., 2015).

### 3.3 Geographical distribution of Hg deposition

**Figure 6:**
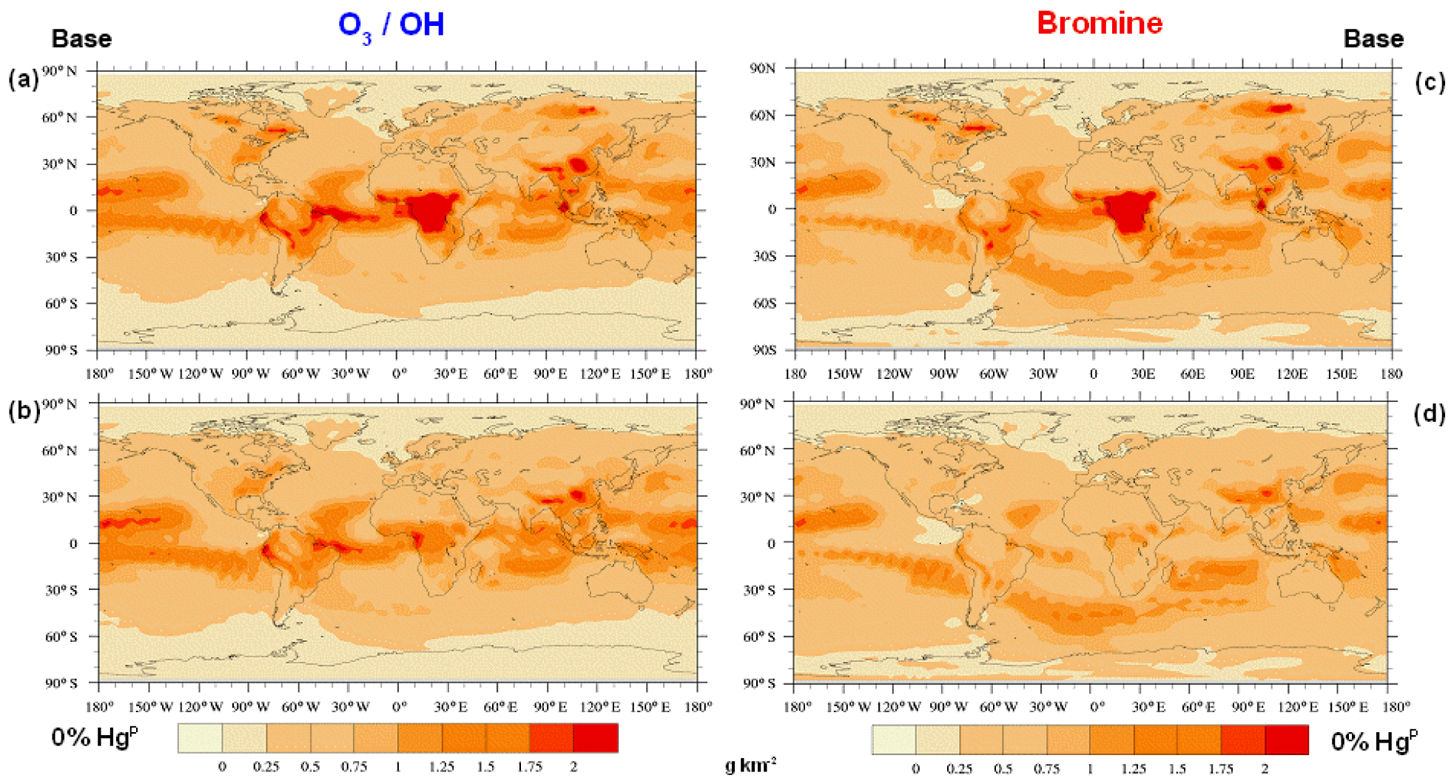
Geographical distribution of the Hg total deposition from model runs including only BB emission sources and assuming two different Hg^P^ emission fractions, 15 % **(a, c)** and 0 % **(b, d)**, for the two oxidation mechanisms considered, O_3_ / OH **(a–b)** and Br **(c–d)**.

**Figure 7:**
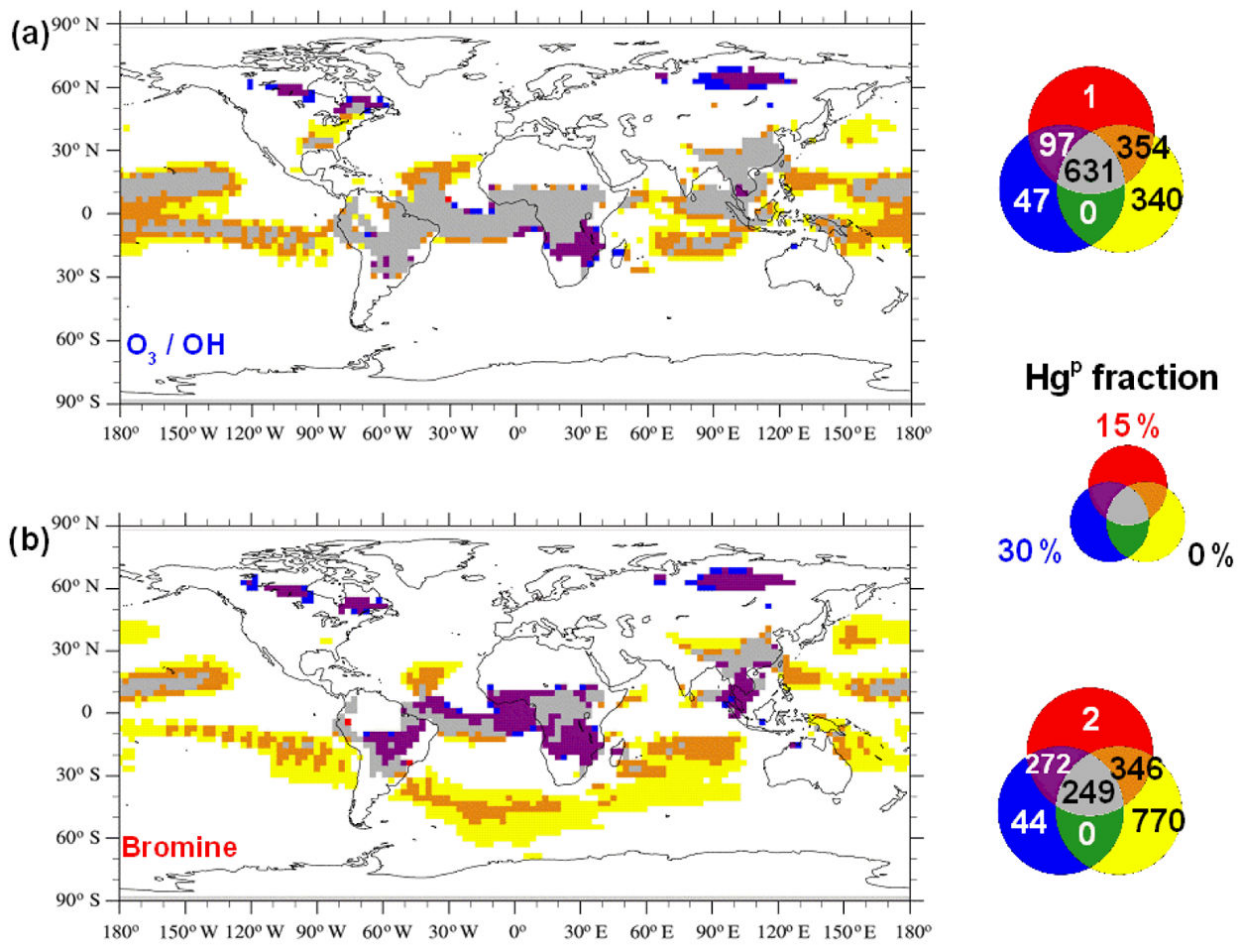
Agreement maps of high Hg deposition model cells obtained considering only BB emissions and assuming 0, 15 and 30 % to be Hg^P^ under both the oxidation mechanisms considered, O_3_ / OH **(a)** and Br **(b)**. The maps show the areas where deposition is greater than *μ* + *σ*.

**Figure 8:**
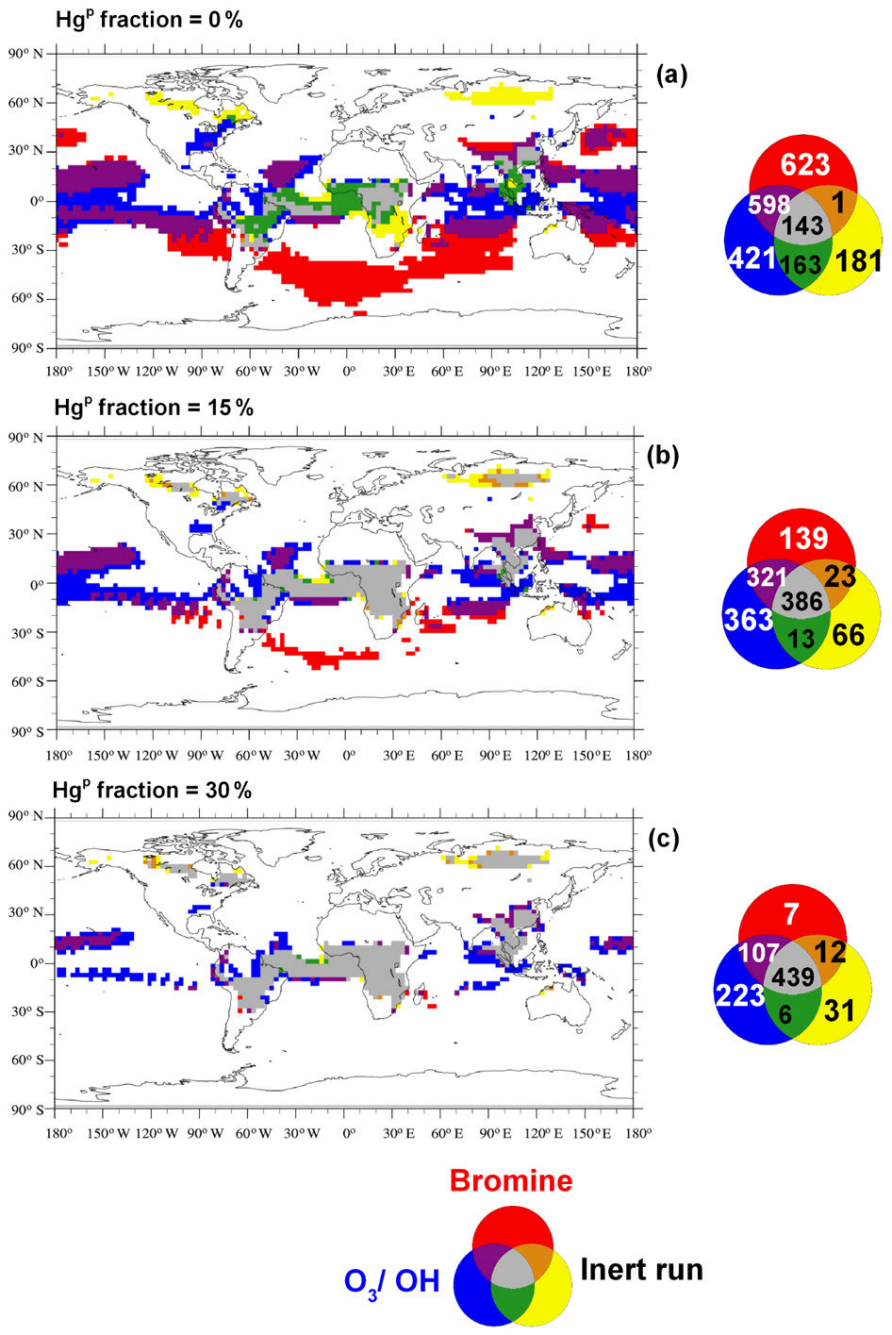
Agreement maps, under three different speciation scenarios, 0 % **(a)**, 15 % **(b)** and 30 % **(c)** Hg^P^, of high Hg deposition model cells obtained considering only BB and using the O_3_ / OH, the Br oxidation mechanisms, and a sensitivity run where all Hg BB emissions were considered inert (i.e. all Hg^P^). The deposition field from for this “inert” run was retained under the three different speciation scenarios. The maps show the areas where deposition is greater than *μ* + *σ*.

Due to the uncertainty in the atmospheric oxidation pathway of Hg, simulations were performed using both O_3_ / OH and Br oxidation mechanisms to investigate their impact on Hg deposition fields. Figure 6a–d compare the geographical distribution of the modelled Hg deposition field using emission fields with 0 % and of 15 % Hg^P^, for each of the oxidation mechanisms. The O_3_ / OH mechanism leads to enhanced deposition in the tropics, whereas the Br mechanism leads to relatively higher deposition over the South Atlantic and Indian oceans. Assuming a fraction of Hg^P^ in the emissions subtracts some ${\text{Hg}}_{\left(g\right)}^{0}$ from the global pool, and this fraction is deposited nearer to emission sources in central Africa, South-East Asia, the Amazon and near the wildfires which occur in North America and in North Asia in the northern hemispheric summer. From Fig. 6, it appears that assuming a fraction of the BB emissions to be Hg^P^ causes the deposition field simulated using the Br oxidation mechanism to more closely resemble that using the O_3_ / OH mechanism. To better understand the combined effect of Hg speciation and oxidation pathway on the deposition distribution, agreement maps were created to highlight the similarities and differences in the distribution of high-deposition (≥*μ* + 1*σ*, the average plus 1 standard deviation) model cells in the different simulations as described in De Simone et al. (2014). Figure 7a and b show the agreement maps of the deposition for three different Hg^P^ fractions using the two oxidation mechanisms. Using the O_3_ / OH mechanism, the number of model cells in which the model predicts high deposition in all three emission speciation scenarios is higher than when using the Br mechanism (631 vs. 248). This is due to the combination of high emissions and high oxidant concentrations in the tropics when using the O_3_ / OH mechanism, constraining Hg deposition to a relatively narrow latitude band. Using the Br mechanism, Hg has a greater possibility of being transported to mid- and high latitudes before being oxidised and deposited. In both the oxidation scenarios the higher deposition over the remote areas of North America and North Asia occurs only when the fraction of Hg^P^ in the emissions is greater than zero. High local contributions to Hg deposition from BB using the Br mechanism occur more frequently when the fraction of Hg^P^ is non-zero (purple in Fig. 7b), un-like the O_3_ / OH simulations. Figure 8 contrasts the results from the two oxidation mechanisms with varying percentages of Hg^P^ and a simulation in which the Hg^P^ fraction was assumed to be 100 %, so that it behaves as an inert tracer. The agreement maps show clearly that the similarity in the deposition fields increases with increasing Hg^P^ fraction, reflected in the number of cells where all three simulations agree (grey in the figure) and the decrease in the number of cells where only one simulation predicts deposition higher than *μ* + *σ* (red, blue and yellow).

### 3.4 Constraints from global measurements networks

**Figure 9:**
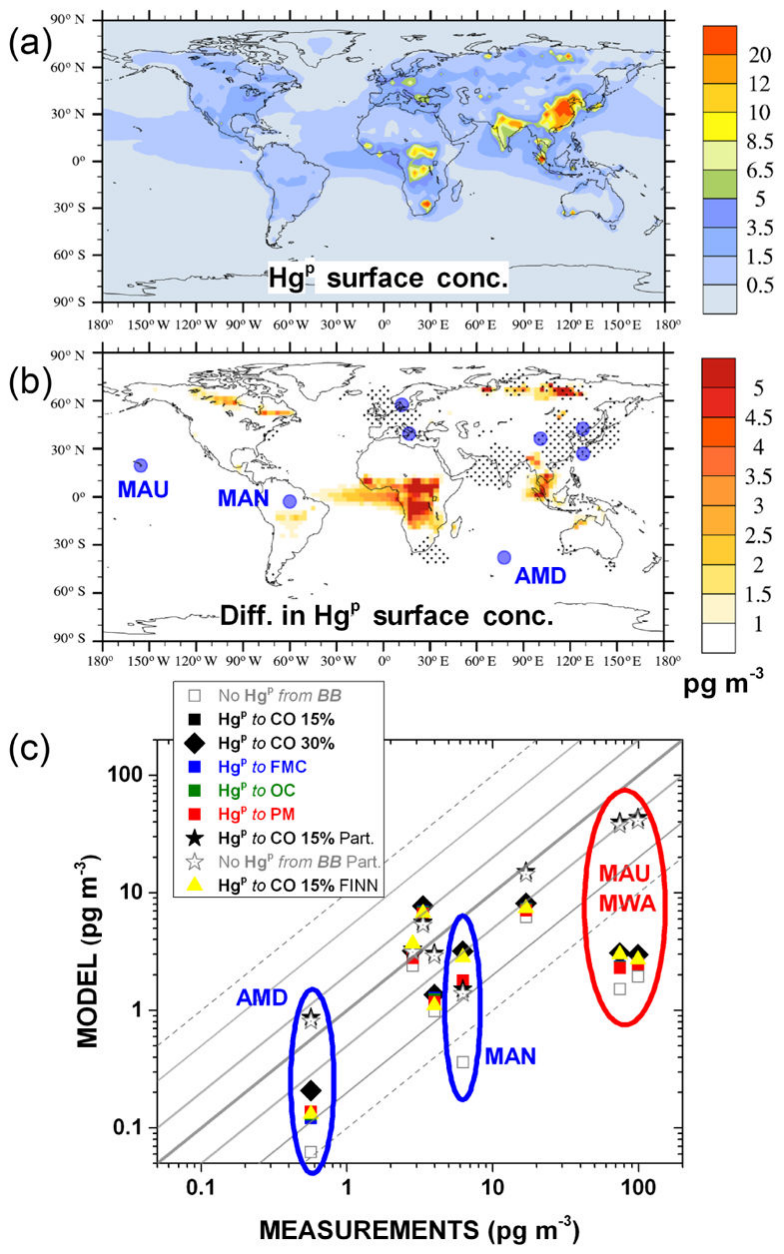
**(a)** Annual averaged surface Hg^P^ concentrations as simulated by BASE run including all emission sources. **(b)** Differences in annual averaged surface Hg^P^ concentrations as simulated by BASE and by NO Hg^P^ runs, both including emissions from all sources. Black dots indicate that differences are not significant based on a Student *t* test at a 95 % confidence interval. Blue bigger points indicate the locations of measurements sites reported in Table 2. Short names are depicted for sites where the differences between BASE and NO Hg^P^ runs are significant. **(c)** Scatter plot of annual averaged Hg^P^ concentrations measured at sites of Table 2 compared with those obtained by different sensitivity runs. The blue circles in the figure indicate values relative to the sites further investigated at an higher temporal resolution (see Fig. 10), whereas the red circles indicate values relative to high-altitude sites affected by processes other than BB.

The output from the simulations including all emissions (as indicated in Table 1) for the year 2013 were compared to measurement data available from GMOS and other monitoring networks. The sites are the same as those used in Travnikov et al. (2016), the measurements from which have been reviewed Sprovieri et al. (2016a, b). Table 6 summarises a selection of metrics from the comparison for total gaseous mercury (TGM; ${\text{Hg}}_{\left(g\right)}^{0}+{\text{Hg}}_{\left(g\right)}^{0}$) and for Hg in wet deposition. The results are in line with those obtained from previous studies (De Simone et al., 2015, 2016) focusing on a different time period, and they indicate a generally good agreement between measured and simulated TGM, especially for the run with the Br-driven oxidation mechanism. For the Hg wet deposition fluxes, the results show poorer performance due to the difficulties for coarse-resolution global models to simulate precipitation events correctly (De Simone et al., 2014; Roeckner et al., 2003). Since the different sensitivity runs considering Hg^P^ from BB differ by a only a small perturbation in the speciation of total Hg emitted from the BASE (or the relevant reference) case, the results are actually indistinguishable from BASE (or the relevant reference) case. Therefore the table reports the comparison only from runs which yield different results. Also, this means that neither wet deposition nor TGM is the most appropriate variable to assess the validity of any of the assumptions concerning Hg^P^ emitted during BB. During 2013, within the GMOS and other Hg monitoring initiatives, a number of measurement sites collected samples of atmospheric Hg^P^. These stations and their precise locations are reported in the Table 2. The result of the comparison with the measurements from these sites is summarised in Fig. 9. Figure 9a shows the annually averaged surface concentrations of Hg^P^ as simulated by the BASE run for 2013. As is evident, surface Hg^P^ hotspots are close to the industrial areas of eastern Europe, India, East Asia and South Africa and to areas characterised by significant BB activity, including Indonesia, central Africa and boreal areas of Canada and Asia.

A first analysis to find those areas where the model run, assuming a fraction Hg^P^ from BB (i.e. BASE), gives results that are statistically distinguishable from the model run assuming Hg from BB to be only ${\text{Hg}}_{\left(g\right)}^{0}$ was performed to identify the measurements sites best suited for further analysis.

**Figure 10:**
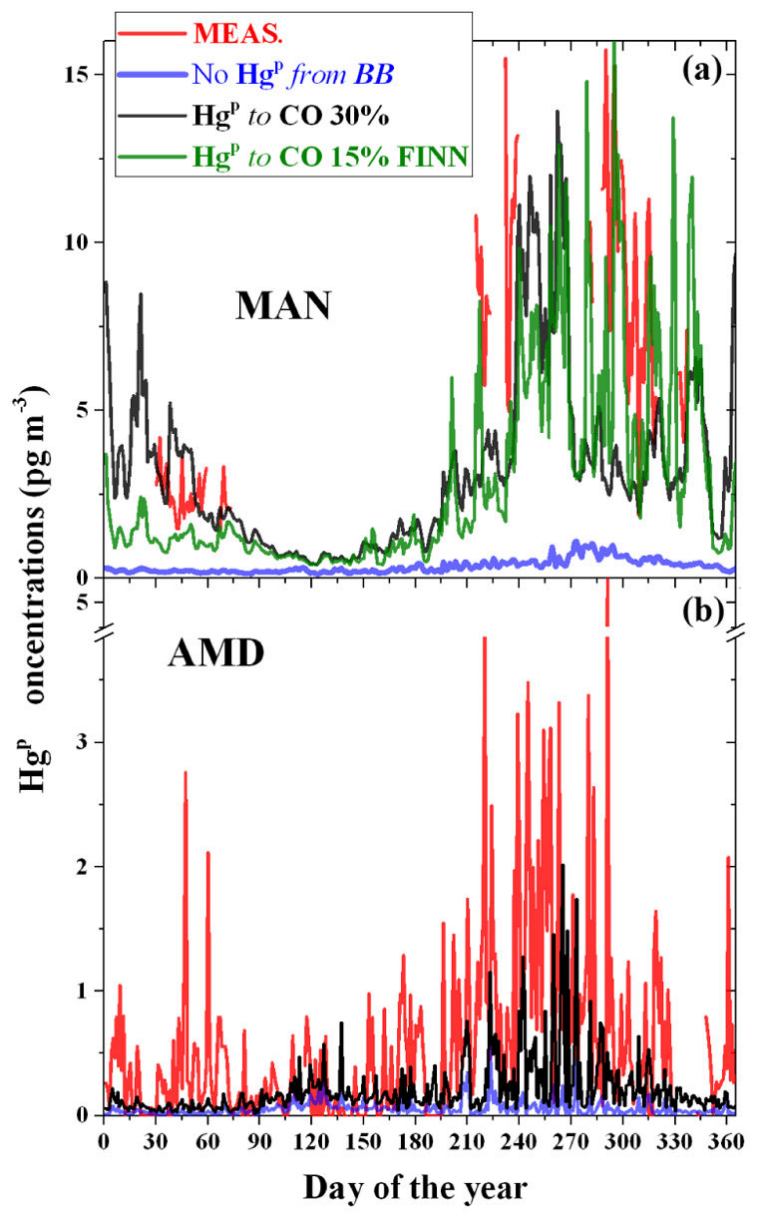
Temporal evolution of daily averaged surface Hg^P^ concentrations measured at Manaus (MAN) and Amsterdam Island (AMD) for the entire 2013, compared with a selection of sensitivity runs.

**Figure 11:**
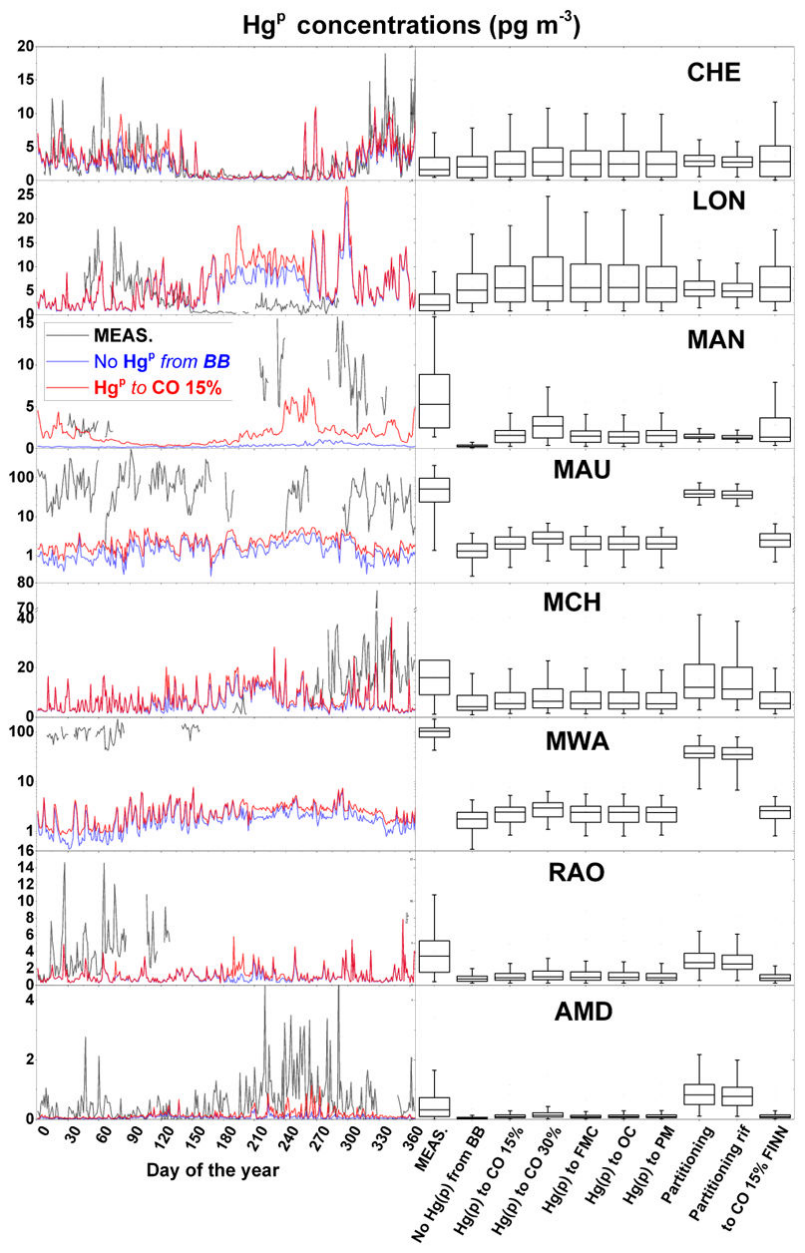
Left column: temporal evolution of the daily averaged surface Hg^P^ concentrations measured at all sites from Table 2 for the entire 2013, compared with the modelled values as simulated by BASE and by NO Hg^P^ runs, including emissions from all sources. Right column: box plots of the distribution of the of the daily averaged surface Hg^P^ concentrations, for the entire 2013, as measured and simulated by the different sensitivity runs. Note the logarithmic for both MAU and MWA subplot.

**Figure 12:**
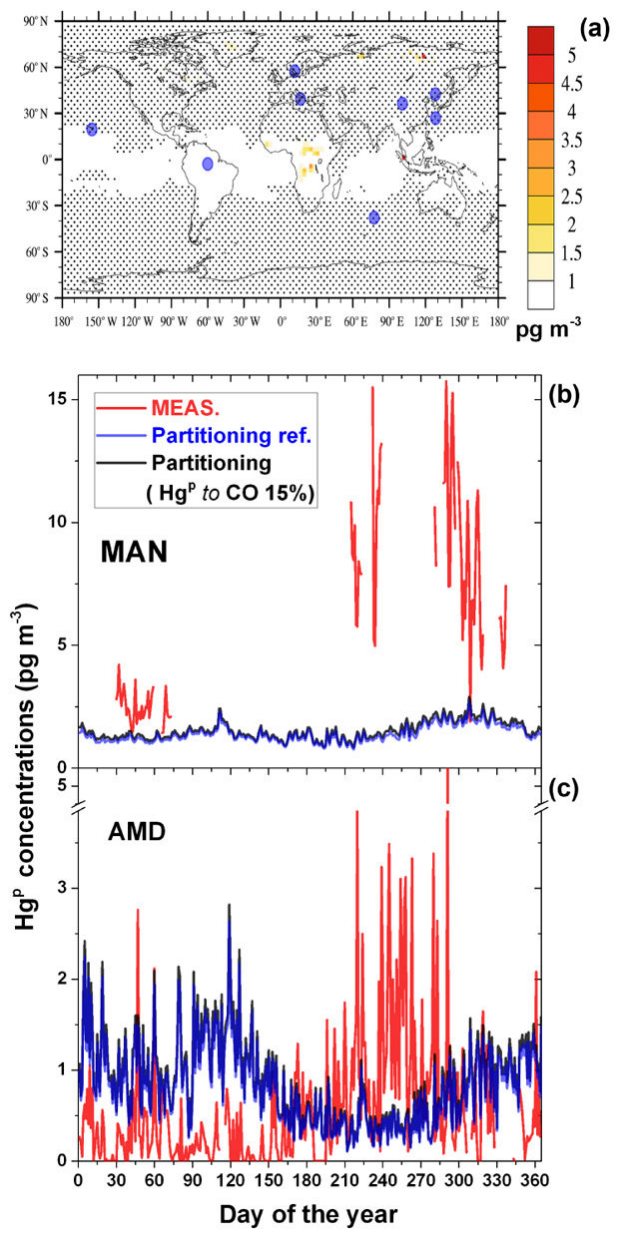
**(a)** Differences in annual averaged surface Hg^P^ concentrations as simulated by Partitioning and by Partitioning ref. runs, both including emissions from all sources and the temperature-dependent Hg^II^ gas-particle partitioning as implemented in Amos et al. (2012). Black dots indicate that differences are not significant based on a Student *t* test at a 95 % confidence interval. Bigger blue points indicate the locations of measurements sites reported in Table 2. Temporal evolution of daily averaged surface Hg^P^ concentrations are measured at Manaus (MAN) and Amsterdam Island (AMD) for the entire 2013, compared with the modelled values from the same sensitivity runs.

The geographical distribution of these differences is reported in panel b of Fig. 9. The areas were the anthropogenic input is the greatest differ little between the simulations (based on a Student *t* test at 95 % level of confidence), as indicated by dot points in the panel. Most of the stations, depicted by the blue solid points in the same panel, are within these regions and therefore unsuitable for the analysis. Only three stations are in areas where the model results are significantly different. These, the short names of which are reported in the panel, are Amsterdam Island (AMD), Manaus (MAN) and Mauna Loa (MAU). However, MAU and Mt. Waliguan (MWA) are high-altitude sites and affected by processes other than BB. For both the remaining stations (AMD and MAN), the fraction of Hg^P^ that is assumed to be emitted by anthropogenic activities, as estimated by AMAP2010 inventory (AMAP/UNEP, 2013), is not sufficient alone to explain the averaged Hg^P^ concentrations collected over the year, as is evident from Fig. 9c. The inclusion of 30 % Hg^P^ from BB emissions at MAN and AMD and also the inclusion of 15 % Hg^P^ from BB as using the FINN inventory at MAN significantly improve the model performances, in terms of the annual average Hg^P^ concentrations. The result of the comparison between the Hg^P^ concentrations collected at these two stations with the same modelled at the same points by a selection of sensitivity runs at an finer temporal resolution (daily averages) is reported in the two panels of Fig. 10. The same comparisons for all the stations, among with the box and whisker plot of distributions of the Hg^P^ concentrations measured and modelled, are reported in Fig. 11. Although the measurement coverage of the year at MAN is sporadic, it is an important station because it is situated in a remote area where the local Hg emissions are due only to ASGM (only ${\text{Hg}}_{\left(g\right)}^{0}$) and BB (Sprovieri et al., 2016b). The consistent reduction of the error between measured and modelled Hg^P^ concentrations when considering a fraction of particulate bound Hg emitted from BB (NRMSE from 48 to 34 % and 27 for 30 % Hg^P^ and FINN, respectively) clearly indicates the role of BB on the observed Hg^P^ values. At AMD (Fig. 10b), the inclusion of the fraction of Hg^P^ from BB results only in a slightly better agreement with the measurements (NRMSE from 16 to 14 %). However, the Hg^P^ event matching grows from 25 to 32 %, especially in the last part of the year. These Hg^P^ events have been associated with BB events in the central Africa in Angot et al. (2014). Peaks was evaluated using the “findpeak” function in MATLAB, available from https://it.mathworks.com/help/signal/ref/findpeaks.html. To summarise, it seems that the emissions of a fraction Hg^P^ from BB is plausible and supported by the measures of atmospheric Hg^P^, at least for the period investigated and for the location of the two remote stations AMD and MAN. However, it has to be noted that the uncertainties related to the precise nature of atmospheric Hg^P^ and to the processes it undergoes in the atmosphere could have an appreciable impact on the model results. For example, the assumption of a temperature-dependent gas-particle Hg^II^ partitioning proposed by Amos et al. (2012) (i.e. the “Partitioning” and “Partitioning ref” runs) yield overall better model agreement with annually average Hg^P^ concentrations (stars in Fig. 9c). However, comparing the modelled daily average time series with measurements results in clearly poorer performance at both the AMD and MAN stations (see Fig. 12b and c). More importantly, this assumption tends to render statistically indistinguishable (Student *t* test at 95 % level of confidence) the contribution of any eventual Hg^P^ from BB, as evident from Fig. 12a.

### 3.5 Uncertainty and biomass burning versus anthropogenic impact

Besides the uncertainty related to the atmospheric Hg oxidation mechanism (Hynes et al., 2009; Subir et al., 2011, 2012; Gustin et al., 2015; Ariya et al., 2015) there are a number of other factors that lead to uncertainty in ascertaining the fate of Hg released by BB. Some of the model assumptions and parametrisations, in particular emission height, made little difference to the eventual deposition fields in the case where emissions from BB were considered to be $100\;\%\;{\text{Hg}}_{\left(g\right)}^{0}$ (De Simone et al., 2015). Other sensitivity studies of the speciation of anthropogenic emissions reveal that varying the fractions of ${\text{Hg}}_{\left(g\right)}^{\text{II}}$ and Hg^P^ can result in quite different Hg deposition patterns due to their shorter residence time compared to ${\text{Hg}}_{\left(g\right)}^{0}$ (De Simone et al., 2016; Bieser et al., 2014).

**Figure 13:**
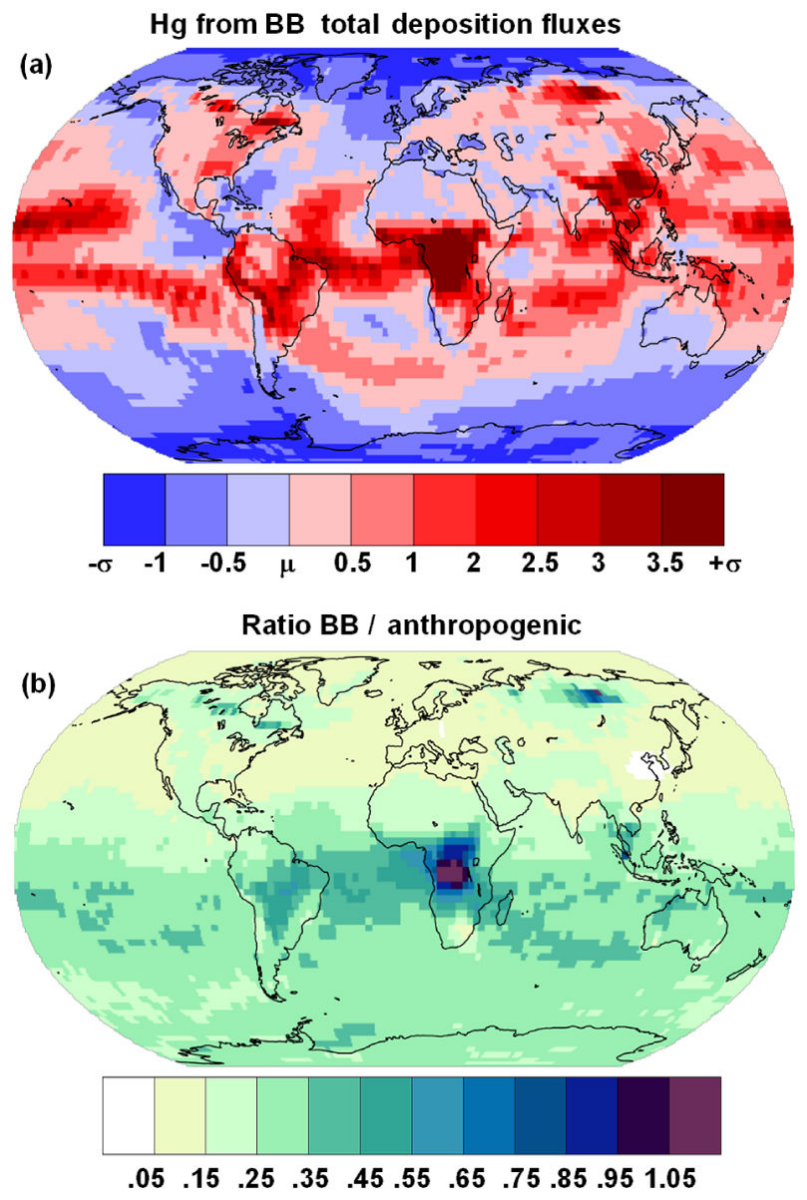
Geographical distribution of the total Hg deposition from BB emissions obtained from an ensemble of simulations for the year 2013 **(a)** in terms of the average (*μ*) and standard deviation *σ* of the ensemble. The comparison of the BB simulation with an ensemble of runs including only anthropogenic emissions (De Simone et al., 2016) shows **(b)** the geographic distribution of the fraction of the BB contribution to the Hg deposition from the anthropogenic sources.

**Figure 14:**
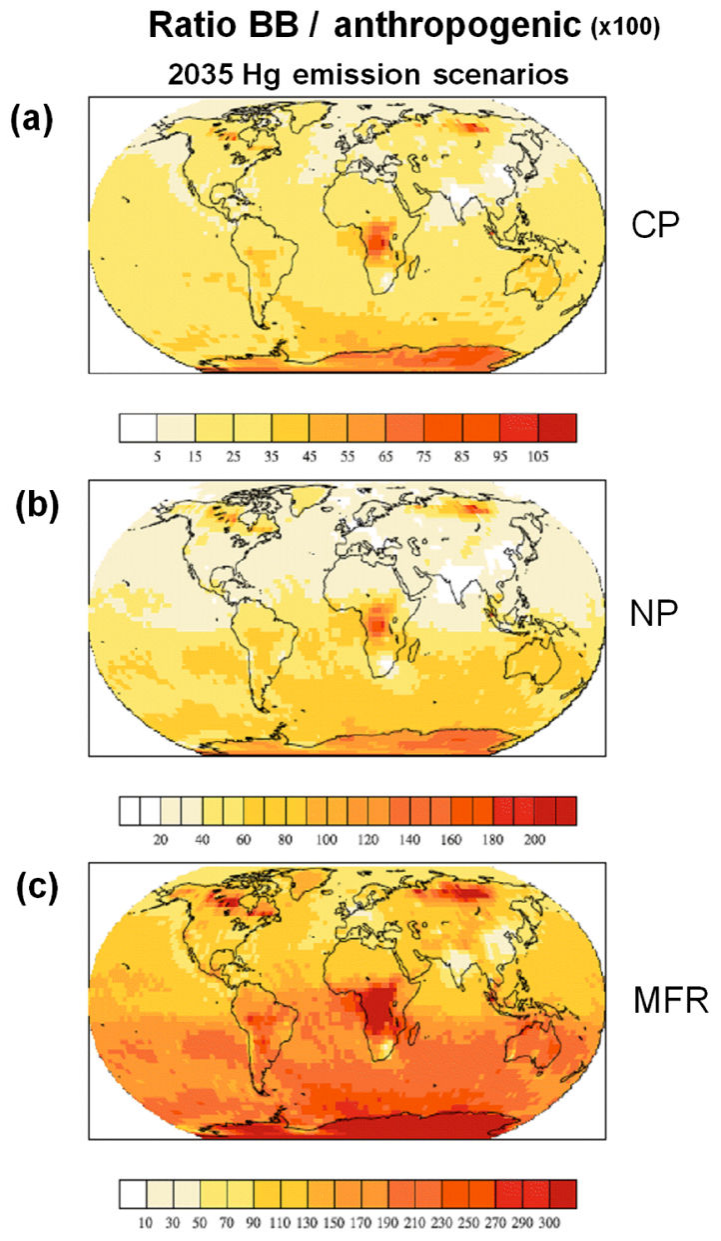
Ratio of the Hg deposition due to biomass burning with respect to Hg deposition due to anthropogenic emissions for three anthropogenic emissions scenarios for 2035: **(a)** current policy (CP), **(b)** new policy (NP) and **(c)** maximum feasible reduction (MFR).

However, the choice of the two main vertical profile of the BB emissions used in this study, also when combined with the temporal resolution of the emissions, actually has little influence on the final Hg deposition fields. Emitting all of the Hg in a single model layer does have an impact. However, these cases are a little speculative, and therefore not included in the final analysis. The factor which has the greatest influence on the Hg deposition pattern is the choice of emission inventory, whereas for a given inventory the most important factors are the fraction of Hg^P^ and the oxidation mechanism, although as seen in Sect. 3.3 the impact of the oxidation mechanism decreases with increasing Hg^P^ fraction. The method of calculating the Hg^P^ fraction has a limited impact on deposition on a global scale, with 66 % of Hg deposited over the oceans, but the regional impact does change. Using FMC to determine the Hg^P^ fraction increases deposition to the Arctic by 16 and 13 % (O_3_ / OH and Br) and to the Southern Ocean by 30 and 25 % (O_3_ / OH and Br); see Table 4. Apart from the polar oceans the oceanic basins, most influenced by the fraction of Hg^P^ in the BB emissions are the North and South Pacific and the Indian ocean. The total deposition to individual basins from the limiting 0 and 30 % Hg^P^ cases is included in Table 4. The horizontal pattern correlation method (Santer et al., 1995, 1996) and the non-parametric Kolmogorov–Smirnov two-sample test were used to assess the differences in the deposition fields obtained from the simulations summarised in Table 1, as in De Simone et al. (2015). The results of the comparison of the simulations with the BASE run are presented in Table 3. The results of the Kolmogorov–Smirnov two-sample test were exploited to construct an inspected ensemble, following the approach of Solazzo and Galmarini (2015) and previously employed in De Simone et al. (2015). The ensemble includes only those simulations with realistic assumptions and deposition fields with little or no probability of belonging to the same distribution. Hg deposition from the resulting ensemble is shown in Fig. 13a. The figure shows how the inclusion of Hg^P^ in the BB emissions causes greater deposition near the hotspots of central Africa, Brazil, South-East Asia, North America and North Asia. Nonetheless approximately 70 % of Hg deposition occurs over the oceans, with the Tropical Atlantic, Tropical Pacific and Indian oceans most impacted (see Table 5). Figure 13b compares the BB ensemble results with an ensemble constructed using only anthropogenic emissions, using the EDGAR (Muntean et al., 2014), AMAP2010 (AMAP/UNEP, 2013) and STREETS (Corbitt et al., 2011) inventories (considering both oxidation mechanisms; see Table 1). It can be seen that the contribution of BB to Hg deposition is close to or greater than that from anthropogenic activities in the areas near the locations of wildfires, central Africa, the Amazon, part of the Southern Atlantic and North Asia. The contribution to Hg deposition from BB relative to anthropogenic emissions is greater than 25 % everywhere in the Southern Hemisphere and exceeds 30 % in the South Pacific and South Atlantic (Table 5). As anthropogenic Hg emissions decline the relative impact of BB Hg will rise, as shown in Fig. 14, where the Hg deposition due to BB is compared with Hg deposition from anthropogenic sources in three different emission scenarios for 2035 (see Pacyna et al., 2016, for details of the emission scenarios).

## 4 Conclusions

That a fraction of Hg^P^ is present in BB Hg emissions has been confirmed by several field measurements (Obrist et al., 2007; Finley et al., 2009), and this fact has been suggested as an explanation of high Hg^P^ observations at a remote site (Angot et al., 2014), but this is the first time it has been included in a model study to assess its effects on a global scale. A previous modelling study assuming emissions from BB to be $100\;\%\;{\text{Hg}}_{\left(g\right)}^{0}$ (De Simone et al., 2015) suggested that as much as 75 % of the Hg emitted by BB was deposited to ocean basins, with global implications for food webs and human health. Including a fraction of Hg^P^ in the BB Hg emissions has an impact on the geographical distribution of the deposition fluxes for the year analysed, reducing input to the global oceans and some high-latitude regions, while enhancing potentially negative effects on ecosystems close to areas where significant BB occurs. The presence of Hg^P^ in the emissions decreases the differences seen in Hg deposition patterns produced by employing different oxidation mechanisms. In the remote areas of North Asia and North America, BB has a strong local impact if the Hg^P^ fraction is non-zero. This latter result is independent of the atmospheric oxidation pathway. In simulations with 30 % Hg^P^ in the BB emissions, deposition over the Arctic increases by 11 % with respect to 0 % Hg^P^ (30 % in the Br simulations) and by 16 % when the Hg^P^ fraction is determined by FMC (37 % in the Br simulation). The fraction of Hg^P^ released from BB while having an impact on the land–sea distribution of global Hg deposition, has a more significant impact in particular regions including the polar regions, the South Atlantic and Pacific and Indian oceans. These results apply for the investigated year (2013) and may differ for other years due to the complex interaction of the numerous factors determining the final fate of Hg. However, few alternatives of analysis period exist due the limited time coverage of global measurement network(s). Indeed the year selected for the analysis allowed for the hypotheses tested in this study to be supported by observations at a number of sites from GMOS, which has extended the observational network in the tropics and the Southern Hemisphere (Sprovieri et al., 2016a, b). The eventual emissions of a fraction of Hg^P^ from BB cannot be evaluated by comparison with observed gaseous atmospheric Hg concentrations or Hg in wet precipitation samples due to the very small impact of Hg^P^ from BB on both the atmospheric burden and wet deposition relative to all other emissions sources (≈ 1–2 %). Conversely, its contribution to atmospheric Hg^P^ is comparable to that of anthropogenic activities and therefore may be investigated. The inclusion in the model run of a fraction of Hg^P^ from BB contributes to better model performances at two remote sites, Manaus and Amsterdam Island. However results are not definitive due to the large uncertainty related to Hg^P^ emissions and transformation processes. Further modelling and more measurement sites, particularly in remote areas, would help reduce some of the uncertainties associated with Hg emissions from BB and constrain these processes. Biomass burning has and will continue to play a significant role in the cycling of legacy Hg, and its relative importance is likely to increase as anthropogenic emissions are reduced and global temperatures rise.

## 5 Data availability

Mercury data discussed in this paper are reported within the GMOS central database and are available upon request at http://sdi.iia.cnr.it/geoint/publicpage/GMOS/gmos_historical.zul.

## Figures and Tables

**Table 1:** Simulations performed.

Name	Inventory (BB emission (Mg))	Full version	Emiss. time res.	Fraction Hg^P^ (%)	Map Hg^P^	Chem. mech.	Vertical profile	Scope
BASE	GFED4.1s (390)	Yes	daily	15	CO	O_3_ / OH	PBL	Reference
3-hourly	GFED4.1s (390)		3 h	15	CO	O_3_ / OH	PBL	Emiss. time resol.
Monthly	GFED4.1s (390)		monthly	15	CO	O_3_ / OH	PBL	Emiss. time resol.
HAM-Profile	GFED4.1s (390)		daily	15	CO	O_3_ / OH	HAM	Vertical profile
Only first level	GFED4.1s (390)		daily	15	CO	O_3_ / OH	1st	Vertical profile
Only PBL level	GFED4.1s (390)		daily	15	CO	O_3_ / OH	level of PBL	Vertical profile
3 h + HAM-prof	GFED4.1s (390)		daily	15	CO	O_3_ / OH	HAM	V. Pr. & E. T. res.
Hg^P^ to PM	GFED4.1s (390)	Yes	daily	15	PM	O_3_ / OH	PBL	Hg^P^ mapping
Hg^P^ to OC	GFED4.1s (390)	Yes	daily	15	OC	O_3_ / OH	PBL	Hg^P^ mapping
Hg^P^ to FMC	GFED4.1s (390)	Yes	daily	variable	CO	O_3_ / OH	PBL	Hg^P^ mapping
NO Hg^P^	GFED4.1s (390)	Yes	daily	0	NA	O_3_ / OH	PBL	Fraction Hg^P^
4 % Hg^P^	GFED4.1s (390)		daily	4	CO	O_3_ / OH	PBL	Fraction Hg^P^
30 % Hg^P^	GFED4.1s (390)	Yes	daily	30	CO	O_3_ / OH	PBL	Fraction Hg^P^
100 % Hg^P^	GFED4.1s (390)		daily	100	CO	None	PBL	Transport Hg^P^
Partitioning	GFED4.1s (390)	Yes	daily	15	CO	O_3_ / OH	PBL	Partitioning Hg^P/II^
Partitioning ref.	GFED4.1s (390)	Yes	daily	0	CO	O_3_ / OH	PBL	Partitioning Hg^P/II^
Reduction	GFED4.1s (390)	Yes	daily	15	CO	O_3_ / OH + Red.	PBL	Chemistry
Br	GFED4.1s (390)	Yes	daily	15	CO	Br	PBL	Chemistry
Br No Hg^P^	GFED4.1s (390)		daily	0	NA	Br	PBL	Chemistry
Br 30 % Hg^P^	GFED4.1s (390)		daily	30	CO	Br	PBL	Chemistry
Br Hg^P^ to OC	GFED4.1s (390)		daily	15	OC	Br	PBL	Chemistry
Br Hg^P^ to FMC	GFED4.1s (390)		daily	variable	CO	Br	PBL	Chemistry
GFAS	GFASv1.2 (150; see Sect. 2.3)		daily	15	CO	O_3_ / OH	PBL	Inventory
GFAS Br	GFASv1.2 (150; see Sect. 2.3)		daily	15	CO	Br	PBL	Chemistry
FINN	FINNv1.5 (550)	Yes	daily	15	CO	O_3_ / OH	PBL	Inventory
FINN Br	FINNv1.5 (550)		daily	15	CO	Br	PBL	Chemistry

AMAPOH	AMAP2010		NA	NA	NA	O_3_ / OH	NA	Ratio to anth. emiss.
AMAPBr	AMAP2010		NA	NA	NA	Br	NA	Ratio to anth. emiss.
EDGAROH	EDGAR2008		NA	NA	NA	O_3_ / OH	NA	Ratio to anth. emiss.
EDGARBr	EDGAR2008		NA	NA	NA	Br	NA	Ratio to anth. emiss.
STREETSOH	STREETS2005		NA	NA	NA	O_3_ / OH	NA	Ratio to anth. emiss.
STREETSBr	STREETS2005		NA	NA	NA	Br	NA	Ratio to anth. emiss.

**Table 2:** Characteristics of ground-based sites measuring Hg^P^.

Long name	Short name	Lat	Long	Elev. (m)
Amsterdam Island	AMD	−37.8	77.58	70
Cape Hedo	CHE	26.86	128.25	60
Longobucco	LON	39.39	16.61	1379
Manaus	MAN	−2.89	−59.97	110
Mauna Loa	MAU	19.54	−155.58	3399
Mt. Changbai	MCH	42.4	128.11	741
Mt. Waliguan	MWA	36.29	100.9	3816
Rao	RAO	57.39	11.91	5

**Table 3:** Horizontal pattern correlation (*R*) and probabilities that the Hg deposition fields of the different runs belong to the same distribution as the BASE run (*P*_KS_). The checks in the ensemble column indicate the inclusion of the respective run in the ensemble in Fig. 13.

	Sim.	*R*	*P*_KS_	Ensemble
Time resolution and vertical profile	3-hourly	1	1	
	Monthly	1	0.99	
	HAM-Profile	1	1	
	3 h + HAM-Profile	1	1	

Hg^P^ mapping	Hg^P^ to PM	1	1	
	Hg^P^ to OC	1	0.42	✓
	Hg^P^ to FMC	0.99	0.45	✓

Hg^P^ fraction	NO Hg^P^	0.94	0.38	✓
	4 % Hg^P^	0.97	0.72	✓
	30 % Hg^P^	0.97	0.5	✓

Inventory	GFAS	0.98	0	✓
	FINN	0.96	0	✓

Oxidation mech. and combination	Br	0.96	0	✓
	Br No Hg^P^	0.81	0	✓
	Br 30 % Hg^P^	0.91	0	✓
	Br Hg^P^ to OC	0.95	0	✓
	Br Hg^P^ to FMC	0.94	0	✓
	GFAS Br	0.94	0	✓
	FINN Br	0.92	0	✓

**Table 4:** Hg deposition (Mg) coming from BB to the oceans as obtained by the different runs for the 2013. The last two columns reports the percentage of the total Hg that deposits over sea and land.

Run	Total deposition/Mg								%	

	N. Atlantic	S. Atlantic	N. Pacific	S. Pacific	Indian Ocean	Med. Sea	Arctic	S. Ocean	Sea	Land
BASE	31.7	32.5	75.3	67.4	45.9	1.1	5.0	2.3	66	34
NO Hg^P^	32.1	32.4	82.0	74.4	48.9	1.2	4.7	2.6	71	29
30 % Hg^P^	31.3	32.5	69.3	61.0	43.2	1.0	5.2	2.0	62	38
Hg^P^ to FMC	31.4	32.1	74.3	66.6	44.7	1.1	5.8	2.3	66	34
Br No Hg^P^	26.6	39.4	75.8	83.0	55.3	1.1	3.7	7.6	74	26
Br 30 % Hg^P^	28.0	36.4	61.7	61.1	44.9	0.9	4.8	4.6	62	38
Br Hg^P^ to FMC	27.3	36.8	66.6	68.8	47.1	1.0	5.6	5.8	66	34

**Table 5:** Mercury deposition (Mg) to the oceans for 2013 from BB and comparison (ratio) with deposition from anthropogenic activities for both oxidation mechanisms.

O_3_ / OH	N. Atlantic	S. Atlantic	N. Pacific	S. Pacific	Indian Ocean	Med. Sea	Arctic	S. Ocean
Only BB	29.8	29.9	72.1	63.0	43.0	1.1	4.7	2.1
Only anthropogenic	144.0	80.0	417.7	206.7	151.3	10.0	34.3	11.0
Ratio	0.21	0.37	0.17	0.31	0.28	0.11	0.14	0.19

Br	N. Atlantic	S. Atlantic	N. Pacific	S. Pacific	Indian Ocean	Med. Sea	Arctic	S. Ocean

Only BB	25.7	34.7	65.1	66.2	46.2	0.9	4.2	5.1
Only anthropogenic	153	85.33	457.3	188.3	140	12.33	34	27.3
Ratio	0.17	0.41	0.14	0.35	0.33	0.08	0.12	0.19

**Table 6:** Comparison of the results of BASE and Br simulations including all emissions sources with observations from measurement networks for 2013.

	Total gaseous mercury				Wet deposition			

	Regression		Stats		Regression		Stats	
	Intercept	Slope	*r*	NRMSE %	Intercept	Slope	*r*	NRMSE %
BASE	0.36	0.62	0.72	10.54	5.84	0.04	0.12	6.89
Partitioning	0.34	0.7	0.73	11.9	3.71	0.03	0.14	4.76
Br	−0.08	0.96	0.74	15.68	7.1	0.08	0.18	9.12

## Appendix A: How Hg emission fields are calculated

### A1 Mapping to CO

When mapped to CO, the emissions of ${\text{Hg}}_{\left(g\right)}^{0}$ were calculated from those of CO using a global averaged ER (1.96 × 10^−7^). These were unchanged in the run assuming Hg emissions from BB to be $100\;\%\;{\text{Hg}}_{\left(g\right)}^{0}$ and divided between Hg^P^ and ${\text{Hg}}_{\left(g\right)}^{0}$ species, with ratios 4 : 96, 15 : 85 and 30 : 70, in mass, in the runs considering the respective constant fractions of Hg^P^. Consequently, the geographical and temporal distributions of ${\text{Hg}}_{\left(g\right)}^{0}$ and Hg^P^ BB emissions follow those of CO. For all cases, the GFEDv4 inventory was used, except for those sensitivity runs performed to test the impact of different inventories, FINNv1.5 and GFAS1.4.

### A2 Mapping to OC

When mapped to OC, geographical and temporal distributions of ${\text{Hg}}_{\left(g\right)}^{0}$ BB emissions, as well as the total Hg emitted, were calculated as described in Appendix A1. The fractioning of Hg emissions, in mass, between Hg^P^ and ${\text{Hg}}_{\left(g\right)}^{0}$ species was assumed to be in the ratio 15 : 85. The Hg^P^ emissions so calculated were then geographically and temporally mapped to those of OC from the GFEDv4 inventory.

### A3 Mapping to PM

This mapping method is similar to the one described in Appendix A2, except for the fact the Hg^P^ temporal and geographical distributions follow those of PM from the GFEDv4 inventory.

### A4 Emissions speciation determination by FMC

When using this procedure for determining the BB emission speciation between ${\text{Hg}}_{\left(g\right)}^{0}$ and Hg^P^, the geographical and temporal distributions of ${\text{Hg}}_{\left(g\right)}^{0}$ and Hg^P^ BB emissions and the total Hg emitted were calculated in the same way as described in Appendix A1. The main difference is in that the fractioning of Hg emissions, in mass, between ${\text{Hg}}_{\left(g\right)}^{0}$ and Hg^P^ species were calculated dynamically using the piece wise linear relationship between fuel moisture content empirically determined by relative figure in Obrist et al. (2007). As a proxy for FMC, we used the monthly averaged vegetation water content derived from passive microwave remote sensing data (Advanced Microwave Scanning Radiometer 2 (ASMR2)), and employing the Land Parameter Retrieval Model (LPRM) available at http://gcmd.nasa.gov/search/Metadata.do?Entry=C1235316240-GES_DISC#metadata.
